# Ultrasonography and sonoelastography in cervical lymph node differentiation: A cross-sectional study

**DOI:** 10.6026/973206300214862

**Published:** 2025-12-15

**Authors:** Harish Shivprasad Gupta, Suresh Kumar Toppo, Rajeev Kumar Ranjan, Anima Ranjni Xalxo, Nisha Rai, Soumik Pal, Riya Agarwal, Mohd Ismail

**Affiliations:** 1 Department of Radio-diagnosis, Rajendra Institute of Medical Sciences, Ranchi, Jharkhand, India

**Keywords:** Cervical lymphadenopathy, ultrasonography, sonoelastography, shear wave elastography, strain elastography, diagnostic accuracy

## Abstract

Cervical lymphadenopathy poses a diagnostic challenge, particularly in distinguishing benign from malignant lymph nodes non-invasively. Therefore, it is of interest to evaluate the diagnostic utility of B-mode ultrasonography, color Doppler, strain elastography and shear wave elastography in 100 patients at a tertiary center in East India. Histopathology served as the reference standard to determine sensitivity, specificity and AUC for each modality. Shear wave elastography, especially perinodal rim stiffness, showed the highest individual accuracy, while the combined B-mode-Doppler-SWE approach demonstrated 94% overall accuracy. Thus, we show that integrating shear wave elastography with conventional ultrasonography significantly enhances differentiation of benign and malignant cervical lymph nodes.

## Background:

Cervical lymphadenopathy is a common clinical condition that can result from various benign and malignant processes [1]. The neck contains approximately two-thirds of the body's total lymph nodes, making it a frequent site for pathological changes [2]. Differentiating between benign and malignant cervical lymph nodes is crucial for appropriate patient management, treatment planning and prognostic assessment [3]. While histopathological examination remains the gold standard for diagnosis, it is an invasive procedure that may not always be feasible or necessary [4]. Conventional B-mode ultrasonography has been widely used to evaluate lymph node characteristics such as size, shape, echogenicity and presence of fatty hilum [5]. Malignant lymph nodes typically exhibit a rounded shape, irregular margins, heterogeneous echotexture and absence of fatty hilum compared to benign nodes [6]. However, these morphological features alone may not provide sufficient diagnostic accuracy, as there can be considerable overlap between benign and malignant conditions [7]. Color Doppler imaging adds functional information by assessing vascularity patterns within lymph nodes [8]. Benign reactive nodes typically show hilar vascularity, while malignant nodes often demonstrate peripheral or mixed vascularity patterns with higher resistive indices [9]. Despite these advances, the diagnostic accuracy of conventional ultrasound techniques remains limited, particularly in differentiating small or early-stage malignant nodes from reactive benign nodes [10]. Sonoelastography, a relatively new ultrasound technique, evaluates tissue stiffness by measuring tissue deformation in response to mechanical compression or acoustic radiation force [11]. This technique includes strain elastography (SE) and shear wave elastography (SWE), both of which have shown promise in differentiating benign and malignant lesions in various organs [12]. Strain elastography provides qualitative or semi-quantitative assessment of tissue stiffness by applying external compression, while shear wave elastography generates quantitative measurements of tissue elasticity in kilopascals (kPa) [13]. Recent studies have demonstrated the potential of sonoelastography in improving the diagnostic accuracy of lymph node evaluation [14]. Lyshchik *et al.* reported that strain elastography achieved 85% sensitivity and 98% specificity in differentiating malignant cervical lymph nodes [15]. Similarly, several studies have shown that shear wave elastography provides quantitative measurements that can effectively distinguish between benign and malignant nodes [16, 17]. However, there is limited research comparing the diagnostic performance of different elastography techniques and their combination with conventional ultrasound parameters in the same patient population. Despite these advances, there remains a research gap regarding the optimal combination of ultrasound parameters for maximum diagnostic accuracy and the comparative performance of different elastography techniques in differentiating benign and malignant cervical lymph nodes, particularly in the Indian population where the spectrum of pathologies may differ from Western countries [18]. Abdel Kerim *et al.* compared the diagnostic performance of strain elastography (SE) and shear wave elastography (SWE) for differentiating benign and malignant lymph nodes. SE showed an overall accuracy of 73.9%, but misclassified 72.7% of lymphomas. In contrast, SWE achieved a higher overall accuracy of 88.7% and improved the diagnostic accuracy for lymphomas from 27.3% (with SE) to 68.2% [19]. Therefore, it is of interest to determine the effectiveness of B-mode ultrasonography, color Doppler imaging and sonoelastography (strain elastography and shear wave elastography) in differentiating benign and malignant cervical lymph nodes with histopathological correlation and to compare the diagnostic accuracy of these individual and combined modalities.

## Materials and Methods:

## Study design and setting:

This cross-sectional study was conducted over 18 months (from April 2022 to September 2023) at the Department of Radiodiagnosis, Rajendra Institute of Medical Sciences (RIMS), Ranchi, a tertiary care center in East India. The study protocol was approved by the Institutional Ethics Committee (Memo No. 128 IEC, RIMS; dated 16/04/2022) and written informed consent was obtained from all participants.

## Sample size calculation:

With an expected prevalence of 80%, 95% confidence interval and 8% precision, the required sample size was approximately 100 participants [19].

## Selection criteria:

## Inclusion criteria:

[1] Adults (≥18 years) with palpable or radiologically detected cervical lymph nodes

[2] Clinical suspicion of malignancy or known benign conditions requiring evaluation

[3] Cervical lymph nodes accessible for ultrasound and elastographic assessment (size ≥3 mm in short axis)

[4] Ability and willingness to provide written informed consent

## Exclusion criteria:

[1] History of neck dissection, recent radiotherapy, or chemotherapy targeting the cervical region

[2] Open wounds, active infections, or severe dermatological conditions over the neck preventing adequate scanning

[3] Severe co-morbidities or unstable clinical condition precluding ultrasound evaluation

[4] Inability to maintain required positioning or cooperate during the procedure

[5] Lack of definitive diagnostic follow-up or inappropriate reference standard

## Imaging techniques and equipment:

All imaging examinations were performed using a Philips Affiniti 70G ultrasound machine equipped with an 8-12 MHz high-frequency linear array transducer. This system uses 'EQ' software for strain elastography and 'ElastQ' software for 2D shear wave elastography.

## B-mode ultrasonography:

The neck region was systematically scanned to locate and assess cervical lymph nodes. In patients with multiple lymph nodes, the most suspicious node on standard ultrasound was selected for detailed evaluation and histopathological correlation.

The following B-mode parameters were evaluated:

[1] Short axis diameter (cutoff value: 8 mm)

[2] Short-to-long axis ratio (cutoff value: 0.6)

[3] Presence or absence of fatty hilum

[4] Echogenicity (homogeneous or heterogeneous)

[5] Margin regularity (regular or irregular)

##  Color doppler imaging:

Color and spectral Doppler assessments were performed to evaluate vascularity within the lymph nodes. The color gain was dynamically adjusted to optimize vessel visualization while preventing color noise from adjacent arterial pulsation.

## Vascular patterns were classified as:

[1] Hilar vascularity or no flow (considered benign)

[2] Peripheral or mixed vascularity (considered malignant)

Resistive index (RI) was measured using spectral Doppler with a gate width of approximately 0.5 mm, maintaining a Doppler angle of less than 60 degrees.

## Strain elastography:

In strain elastography, an ultrasound probe applies mechanical force to a lymph node to assess its tissue stiffness. The images are acquired in real time and images with inadequate compression (indicated by red or yellow on a sidebar) are excluded. The resulting patterns are then evaluated and classified on a four-point scale based on a study by Furukawa *et al.* Lymph nodes with type 1 and 2 patterns are considered benign, while those with type 3 and 4 patterns are classified as malignant. Mechanical force was applied to the cervical lymph nodes using the ultrasound probe. Real-time elastographic images were acquired to assess tissue stiffness. Compression and gradual release were performed, with adequate compression indicated by green color on the sidebar. Images with inadequate compression (red or yellow color) were excluded.

## The elastographic patterns were classified into four groups according to Furukawa *et al.* [20]:

[1] Pattern 1: 80% or more of the cross-sectional area is red or green (soft)

[2] Pattern 2: 50% to less than 80% is red or green

[3] Pattern 3: 50% to less than 80% is blue

[4] Pattern 4: 80% or more of the cross-sectional area is blue (hard)

Patterns 1 and 2 were classified as benign, while patterns 3 and 4 were considered malignant. Strain ratio was calculated using - two regions of interest (ROIs) were drawn within the lymph node and an adjacent normal muscle instead at the same depth. The strain ratio was then automatically computed by the US machine [15].

## Shear wave elastography:

Patients were instructed to hold their breath to minimize neck movements during image acquisition. Only elastograms with a confidence threshold of ≥50% were considered. The color map was adjusted so that hard areas appeared red and soft areas appeared blue. Images were displayed in split-screen mode, with gray-scale ultrasound images on the left and elastogram images on the right. Three circular regions of interest (ROIs), each 2 mm in diameter, were placed within the node and the system automatically computed the mean kilopascal (kPa) values and shear velocity for each region. The maximum EQI value among the three ROIs was recorded.

## To calculate the shear elasticity ratio, three additional ROIs were placed on:

[1] The hardest portion within the lymph node

[2] The perinodal rim tissue

[3] The adjacent fatty or muscular tissue

The elasticity ratio was determined by dividing the kPa value of the hardest intra-nodal region by that of the surrounding tissues.

## Histopathological examination:

All participants underwent ultrasound-guided fine-needle aspiration cytology (FNAC), core biopsy, or surgical excision of the cervical lymph nodes. Histopathological examination was performed by experienced pathologists blinded to the imaging results and the final diagnosis (benign or malignant) was documented.

## Statistical analysis:

Data were analyzed using SPSS software (version 26.0, IBM Corp., Armonk, NY, USA).

## Results:

The study included 100 patients with cervical lymphadenopathy, comprising 65 (65%) benign and 35 (35%) malignant cases based on histopathological examination. The mean age of patients with malignant cases (49.26 ± 7.27 years) was significantly higher than those with benign cases (33.77 ± 6.76 years, p < 0.001). There was no statistically significant difference in gender distribution between benign and malignant cases (p = 0.099), although benign cases were more common in males (70.8%) compared to malignant cases (54.3%). Among benign cases, 80% were non-specific lymphadenopathy, while tuberculosis (10.8%) and granulomatous inflammation (9.2%) were less frequent. Malignant cases consisted primarily of thyroid carcinoma (34.3%), oral carcinoma (28.6%) and lymphoma (17.1%), with breast carcinoma (11.4%) and other malignancies (8.6%) comprising the rest. Malignant lymph nodes exhibited significantly larger short axis diameters (10.91 ± 2.79 mm vs. 6.46 ± 2.29 mm, p < 0.001) and higher short-to-long axis ratios (0.89 ± 0.34 vs. 0.55 ± 0.39, p < 0.001) compared to benign nodes. The absence of fatty hilum was significantly more common in malignant nodes (71.4% vs. 29.2%, p < 0.001). Heterogeneous echogenicity (65.7% vs. 38.5%, p = 0.009) and irregular margins (62.9% vs. 38.5%, p = 0.020) were also more frequently observed in malignant nodes. Among individual B-mode parameters, short axis diameter demonstrated the highest diagnostic accuracy (73%), followed by short-to-long axis ratio (72%), presence/absence of fatty hilum (71%), margin regularity (62%) and echogenicity (63%). When combined B-mode parameters were evaluated with any three positive findings considered malignant, the sensitivity was 91.43%, specificity was 92.31% and overall accuracy was 92%. Color Doppler vascularity patterns showed significantly different distributions between benign and malignant cases (p < 0.001). The majority of benign cases (72.3%) exhibited absent or hilar vascularity, while malignant cases predominantly showed mixed or peripheral vascularity (77.1%). Color Doppler imaging demonstrated a sensitivity of 77.14%, specificity of 72.31% and diagnostic accuracy of 74%. Spectral Doppler parameters revealed significantly higher resistive indices in malignant nodes (0.93 ± 0.23 vs. 0.54 ± 0.22, p < 0.001). Using a cutoff value of ≥0.70, spectral Doppler imaging achieved a sensitivity of 74.3%, specificity of 70.8% and diagnostic accuracy of 72% (Table 1 - see PDF, 2 - see PDF).

Malignant nodes exhibited significantly higher strain ratios compared to benign nodes (2.38 ± 0.41 vs. 1.59 ± 0.51, p < 0.001). Using a strain ratio cutoff of ≥2.27, strain elastography demonstrated a sensitivity of 85.7%, specificity of 78.5% and overall accuracy of 81%. When classified by elastography pattern, type 3 and 4 patterns (considered malignant) showed a sensitivity of 82.86%, specificity of 84.62% and accuracy of 84%. Malignant nodes showed significantly higher stiffness values on shear wave elastography compared to benign nodes. The mean shear score of the node was 80.86 ± 20.34 kPa in malignant nodes versus 30.43 ± 9.16 kPa in benign nodes (p < 0.001). The shear score of the perinodal rim was also significantly higher in malignant nodes (39.26 ± 3.11 kPa vs. 27.69 ± 4.33 kPa, p < 0.001). Using a cutoff value of ≥45.50 kPa for the shear score of the node, the sensitivity was 91.4%, specificity was 86.2% and diagnostic accuracy was 88%. The shear score of the perinodal rim with a cutoff of ≥33.50 kPa demonstrated perfect sensitivity (100%), specificity of 86.2% and accuracy of 91%. The elasticity shear ratio was significantly higher in malignant nodes (2.53 ± 0.50 vs. 2.17 ± 0.47, p < 0.001). With a cutoff of ≥2.39, the elasticity shear ratio showed a sensitivity of 71.4%, specificity of 78.5% and accuracy of 76%. The combination of B-mode, color Doppler and strain ratio (BM+CD+SR) yielded a sensitivity of 77.14%, perfect specificity of 100% and overall accuracy of 92%. The combination of B-mode, color Doppler and shear score of the node (BM+CD+shear score) achieved the highest diagnostic performance with a sensitivity of 82.86%, specificity of 100% and accuracy of 94%. The combination of B-mode, color Doppler and elasticity shear ratio (BM+CD+ESR) showed a sensitivity of 62.86%, specificity of 100% and accuracy of 87. Figure 1 (see PDF) illustrates the diagnostic accuracy of various ultrasonographic parameters in lymph node evaluation, showing that individual features such as margin regularity (0.62), echogenicity (0.63) and fatty hilum presence (0.71) have relatively low accuracy, while parameters like strain ratio (0.81) and shear score of node (0.88) perform better. However, the highest accuracy is achieved with combined approaches, particularly when B-mode, Color Doppler and Elastography techniques are integrated, with BM+CD+Elasticity Shear Ratio reaching 0.97, highlighting that multimodal ultrasound assessment is far superior to single-parameter evaluation in differentiating lymph node pathology (Table 3 - see PDF).

## Discussion:

This study evaluated the diagnostic performance of B-mode ultrasonography, color Doppler imaging and sonoelastography in differentiating benign and malignant cervical lymph nodes. Our findings demonstrate that the combination of these modalities, particularly B-mode, color Doppler and shear wave elastography, provides excellent diagnostic accuracy, reaching 94% when using the shear score of the node. The demographic characteristics of our study population revealed that malignant nodes were more prevalent in older patients, with a mean age of 49.26 years compared to 33.77 years for benign cases. This age-related difference is consistent with previous studies and reflects the known epidemiological trend that the risk of malignancy increases with age [21]. While there was no statistically significant difference in gender distribution, we observed a trend toward a higher proportion of females in the malignant group, which may be related to the higher incidence of thyroid and breast cancers in women and their potential for cervical lymph node metastasis [22]. Our histopathological analysis showed a diverse spectrum of diagnoses, with non-specific lymphadenopathy being the most common benign condition and thyroid and oral carcinomas being the predominant malignant causes. This distribution is consistent with the epidemiological patterns reported in other Indian studies, although the proportion of tuberculous lymphadenitis in our study (10.8%) was lower than that reported in some previous Indian studies, which may reflect regional variations or referral patterns [23]. Among individual B-mode parameters, the short axis diameter demonstrated the highest diagnostic accuracy (73%), followed by the short-to-long axis ratio (72%). These findings are consistent with established criteria emphasizing size and shape as key differentiating features [24]. However, the moderate sensitivity and specificity of individual B-mode parameters in our study (ranging from 62% to 73%) highlight the limitations of relying solely on morphological features, similar to findings reported by Gupta *et al.* [25]. Color Doppler imaging provided valuable functional information, with malignant nodes showing significantly higher resistive indices and predominantly peripheral or mixed vascularity patterns. Our findings of 77.14% sensitivity and 72.31% specificity for color Doppler imaging are comparable to those reported by Vineela *et al.* [26] but lower than the 92% accuracy reported by Somali *et al.* [27]. These discrepancies may be attributed to differences in patient populations, vascular pattern definitions and operator experience.

Sonoelastography, particularly shear wave elastography, demonstrated superior diagnostic performance among individual parameters in our study. The shear score of the perinodal rim achieved perfect sensitivity (100%) with 86.2% specificity and 91% accuracy, while the shear score of the node showed 91.4% sensitivity, 86.2% specificity and 88% accuracy. These findings are consistent with previous studies reporting high stiffness values in malignant lymph nodes [28, 29]. The excellent performance of perinodal rim assessment may be related to the perinodal fibrosis and tumor infiltration that often occurs in malignant nodes, which increases tissue stiffness in this region [30]. Strain elastography also demonstrated good diagnostic performance in our study, with a sensitivity of 85.7%, specificity of 78.5% and accuracy of 81% using a strain ratio cutoff of ≥2.27. These results are comparable to those reported by Lyshchik *et al.* who found 85% sensitivity and 98% specificity for strain elastography [31]. However, shear wave elastography outperformed strain elastography in our study, which may be attributed to its quantitative nature and reduced operator dependence [32]. The combination of multiple imaging modalities significantly improved diagnostic accuracy in our study. The combination of B-mode, color Doppler and shear wave elastography (node shear score) achieved the highest overall accuracy of 94%, with perfect specificity (100%). This multi-parametric approach leverages the strengths of each technique while compensating for their individual limitations, similar to findings reported by Yang *et al.* [33]. The perfect specificity of this combination suggests that when all three modalities indicate malignancy, the likelihood of a false positive result is extremely low, which could be particularly valuable in guiding clinical decision-making. Our study has several strengths, including its prospective design, comprehensive evaluation of multiple imaging parameters and histopathological correlation. However, some limitations should be acknowledged. The sample size, while adequate for statistical analysis, was relatively small and may limit the generalizability of our findings. Operator dependency in ultrasound techniques, particularly in strain elastography, is another potential limitation, although we attempted to standardize procedures as much as possible. Additionally, the specific types of malignancies encountered in our study population may influence the results and further studies with larger, more diverse samples are needed to validate our findings. Future research should focus on standardizing elastography protocols and establishing optimal cutoff values for different patient populations. Additionally, studies comparing the cost-effectiveness of multi-parametric ultrasound approaches versus other imaging modalities such as contrast-enhanced CT or MRI would be valuable in guiding clinical practice.

## Conclusion:

The combination of B-mode ultrasonography, color Doppler imaging and shear wave elastography provides excellent diagnostic accuracy in differentiating benign from malignant cervical lymph nodes. Among individual parameters, shear wave elastography, particularly assessment of the perinodal rim, showed the highest diagnostic performance. The multi-parametric approach significantly improved diagnostic accuracy compared to individual modalities, highlighting the value of comprehensive ultrasound evaluation in the assessment of cervical lymphadenopathy.
